# Th17-Immune Response in Patients With Membranous Nephropathy Is Associated With Thrombosis and Relapses

**DOI:** 10.3389/fimmu.2020.574997

**Published:** 2020-11-26

**Authors:** Marion Cremoni, Vesna Brglez, Sandra Perez, Fabrice Decoupigny, Kévin Zorzi, Marine Andreani, Alexandre Gérard, Sonia Boyer-Suavet, Caroline Ruetsch, Sylvia Benzaken, Vincent Esnault, Barbara Seitz-Polski

**Affiliations:** ^1^Service de Néphrologie-Dialyse-Transplantation, CHU de Nice, Université Côte d’Azur, Nice, France; ^2^Unité de Recherche Clinique de la Côte d’Azur (UR2CA), Université Côte d’Azur, Nice, France; ^3^Centre de Référence Maladies Rares Syndrome Néphrotique Idiopathique, CHU de Nice, Université Côte d’Azur, Nice, France; ^4^UMR ESPACE 7300 CNRS, Université Côte d’Azur, Nice, France; ^5^Laboratoire d’Immunologie, CHU de Nice, Université Côte d’Azur, Nice, France

**Keywords:** membranous nephropathy, inflammation, Th17-profile, fine air particulate matter, thrombosis, relapse, non-invasive biomarker, prognosis

## Abstract

Membranous nephropathy (MN) is a rare autoimmune kidney disease. Most autoimmune diseases are associated with a pro-inflammatory Th17-immune response, but little is known about immune dysregulation in MN. In China, MN was associated with exposure to fine air particulate matter (PM_2.5_) that could act as a danger signal and redirect immune response toward the Th2 or Th17 pathway. We aimed to analyze the cytokine profile of MN patients and to study the possible environmental factors involved in this immune reorientation, as well as the consequences on the prognosis of the disease. In this prospective study, 59 MN patients filled a comprehensive lifestyle questionnaire. Peripheral blood cells from MN patients were stimulated *in vitro* to measure the cytokines produced in supernatant. Cytokine profiles of MN patients were compared to 28 healthy donors and analyzed regarding individual PM_2.5_ exposure. Compared to healthy donors, MN patients had higher serum levels of Th17 and Th2 cytokines IL-17A (62 pg/ml [IQR, 16–160] versus 31 [IQR, 13–51], *P*=0.035), IL-6 (66767 pg/ml [IQR, 36860–120978] versus 27979 [IQR, 18672–51499], *P*=0.001), and IL-4 (12 pg/ml [IQR, 0–33] versus 0 pg/ml [IQR, 0–0], *P*=0.0003), respectively, as well as a deficiency of Th1 and regulatory T cell cytokines IFN-γ (5320 pg/ml [IQR, 501–14325] versus 18037 [IQR, 4889–31329], *P*=0.0005) and IL-10 (778 pg/ml [IQR, 340–1247] versus 1102 [IQR, 737–1652], *P*=0.04), respectively. MN patients with high IL-17A levels lived in areas highly exposed to PM_2.5_: 51 μg/m^3^ versus 31 μg/m^3^ for patients with low IL-17A levels (*P*=0.002) while the World Health Organization recommends an exposition below 10 μg/m^3^. MN patients with Th17-mediated inflammation had more venous thromboembolic events (*P*=0.03) and relapsed more often (*P*=0.0006). Rituximab treatment induced Th1 and regulatory T cell cytokines but did not impact Th17 cytokines. MN patients with Th17-mediated inflammation which appears to be related to an urban environment have worse prognosis. Alternative strategies targeting dysregulated cytokine balance could be considered for these patients at high risk of relapse.

## Introduction

For several decades, Western countries have been facing an increasing incidence of allergies and autoimmune diseases. Immune-mediated conditions are thought to result from a complex interplay between genetic predisposition, immune dysregulation, and environmental factors (1). Membranous nephropathy (MN) is a rare autoimmune disease (incidence 1.3 cases per 100 000 inhabitants affecting more men than women) (2) with an increasing prevalence (3), characterized by subepithelial immune deposits containing IgG and complement fractions with alteration of the glomerular basement membrane structure (4, 5) related to autoantibodies against podocyte proteins: M-type phospholipase A2 receptor 1 (PLA2R1) or thrombospondin type-1 domain-containing 7A (THSD7A) in 70% and 3% of patients, respectively (6–9). Anti-PLA2R1 and anti-THSD7A antibodies titers correlate with disease activity and predict disease outcome (10, 11). Disease evolution is highly variable with spontaneous remission, persistent proteinuria or end-stage kidney diseases (ESKD) (12). Rituximab is a first line anti-CD20 immunosuppressive drug often used to treat MN patients which induces remission in about 35% to 67% of cases (13–15), while MN recurs after remission in about 20% of cases (14). Nephrotic syndrome has been correlated with an exceptionally high risk of venous thromboembolic events (16, 17).

In spite of these advances in diagnosis, prognosis and treatment, little is known about the risk factors leading to the onset of MN. Several genome-wide association studies identified alleles at two genomic loci associated with idiopathic MN: HLA-DQA1 and single-nucleotide polymorphisms (SNPs) on PLA2R1 (18–26). More recently, a novel genome-wide significant (GWAS) risk locus for MN with large effects encoding two transcriptional master regulators of inflammation, NFKB1 and IRF4, has been discovered suggesting MN is an inflammatory disease (27).

Little is known about immune dysregulation in MN. While Th1 is usually associated with intracellular pathogens (Th1 cells secrete IFN-γ), Th2 is activated by parasites or allergens and secrete IL-4 and IL-5 (28). Th17-immune response is a pro-inflammatory immune pathway associated with autoimmune diseases (29, 30). Th17 cells require specific cytokines, such as transforming growth factor-β (TGF-β) combined with IL-6 or IL-21 for their differentiation (31). Th17 cells secrete a characteristic profile of cytokines including IL-17A, IL-17F, IL-21, and IL-22, which recruit and activate neutrophils and macrophages to fight against extracellular pathogens or mediate the development of autoimmune diseases (32). Th17 and regulatory T cells (Treg cells) are two subsets with opposite actions (33). They play an important role in the prevention of autoimmunity and in the regulation of immune responses against infections and cancer in a cell-contact-dependent manner or by secreting inhibitory cytokines like IL-10 or TGF-β (34).

In MN, several studies led to different conclusions regarding the predominant type of immune response. Kuroki et al. showed 15 years ago that MN is associated with a Th2-type immune response with increased IL-4 levels (35–37) using real time PCR. Recently, Rosenzwajg et al. and Roccatello et al. described a low level of Treg cells in two cohorts of MN (n=25 and n=17, respectively) at diagnosis and an increasing level of these cells after remission induced by rituximab (38, 39), associated with low levels of a Treg cytokine IL-35 that also increases with remission (38). Moreover, Rosenzwajg et al. did not identify cytokine profiles in non-stimulated cells with a decrease of IL-17A in MN patients compared to healthy donors (39), which could be explained by a cytokine discharge in urine due to high proteinuria. Very recently, Li et al. showed an increased number of Th17 cells in primary MN patients after a stimulation with leukocyte activation cocktail (BD GolgiPlug™) using a flow cytometry-based intracellular cytokine detection method, and an increased plasma level of Th17 cytokines by ELISA (40).

Environmental factors may play a role in the pathophysiology of MN. The incidence of post-infectious MN is declining in industrialized countries, in part due to vaccination campaigns against hepatitis B, while its autoimmune form is stable or even increasing (41–44), which could be explained by hygiene hypothesis: low exposure to infectious agents in childhood induces a Th1/Th2 or Th17 imbalance causing allergic and autoimmune diseases. Environmental factors (pollution, vitamin D deficiency, smoking) could act as danger signals and redirect immune response toward the Th2 or Th17 pathway (1, 45). In China, Xu and al. showed a geographical correlation between the occurrence of MN (399 MN diagnosed in 2014) and the satellite analysis of exposure to fine particles (PM_2.5_) detected over the same period (3).

We aimed to clarify which immune response plays a major role in MN. We analyzed the cytokine profile in the supernatant of peripheral blood cells from MN patients after a non-specific stimulation of immune cells and sought to establish whether this profile could be related to the environmental data collected.

## Materials and Methods

### Study Design and Population

We performed a prospective cohort study in the Department of Nephrology at Nice University Hospital. The inclusion criteria were: 1) patients with biopsy proven MN or with either anti-PLA2R1- or anti-THSD7A-associated MN, at diagnosis or at relapse; 2) urine protein/creatinine ratio (UPCR)>1 g/g and immunological activity (for PLA2R1- or THSD7A-related MN); 3) not having received immunosuppressive therapy in the 6 months before inclusion; 4) followed in the Department of Nephrology at Nice University Hospital between January 2016 and January 2018; 5) ability to sign an informed consent. Fifty-nine MN patients were recruited, and for 26 of them with active disease at inclusion (UPCR>3.5g/g) an additional follow-up sample at complete or partial remission was collected. Complete remission was defined as proteinuria <0.3g per 24 h and partial remission as proteinuria >0.3 but <3.5g per 24 h or a decrease in proteinuria by at least 50% from the initial value and <3.5g per 24 h, according to international Clinical Practice Guidelines (KDIGO) (46). Healthy donors of the same age and living in the same environment were also recruited. Patients and healthy donors with infection or breastfeeding at the time of sampling were excluded.

The study protocol conformed to the ethical guidelines of the 1975 Declaration of Helsinki and was approved by the appropriate institutional review committee (NCT02199145). Written informed consent was obtained from participants prior to inclusion in the study.

### Blood Collection and Cytokine Assay

One milliliter of whole blood was collected and stimulated with immune ligands (anti-CD3 as T-cells stimulant, R848 as TLR 7/8 agonist, or both) on single lyophilized spheres (LyoSphere™, Qiagen) within 8 h from blood collection. Stimulated blood samples were incubated for 16 to 24 h at 37°C and then centrifuged at 2,000 to 3,000 x g for 15 min to harvest the stimulated serum. Stimulated serum was stored at −20°C until the analysis and freeze-thaw cycles were minimized to preserve the quality of the samples. Serum levels of 11 cytokines (IL-17A, IL-6, IL-1β, IFN-γ, IL-12p70, TNF-α, IL-10, IL-5, IL-4, IL-13, and GM-CSF) were measured using the ProcartaPlex™ Immunoassay Kit (Luminex™, ThermoFisher) or the custom-designed cartridges Ella (ProteinSimple™), following the manufacturers’ instructions. ProcartaPlex™ Immunoassays incorporate magnetic microsphere technology to enable simultaneous detection and quantification of multiple cytokines in serum, and Ella measures cytokines in a microfluidic Simple Plex cartridge. All samples were measured both as pure and diluted 1:100, as the detection threshold differs among cytokines.

### Data Collection

Clinical and environmental data were collected during the medical consultation, using the patient’s medical record and *via* a questionnaire which focused on lifestyle, usual area of residence and medical history before the first symptoms of MN.

Exposure level to air pollutants (PM_2.5_, PM_10_, NO_X_, CO, SO_2_, and C_6_H_6_) was publicly available (Atmosud (a French public institution)). We used cumulative exposition during the year before the diagnosis or the relapse measured by Atmosud using sensors present at different points in the region studied (**Supplemental Figure 1**).

### Statistics

For descriptive statistics, data are presented as mean and standard deviation for quantitative variables with Gaussian distribution, as median and range for quantitative variables with non-Gaussian distribution, or as numbers and percentages for qualitative variables. The Shapiro-Wilk test was used to determine if a variable had a Gaussian distribution or not. Quantitative variables were compared by the unpaired *t*-test or one-way ANOVA if the values were normally distributed and by the Mann-Whitney test if they were not. Qualitative variables were compared using Chi-square test or Fisher’s exact test as appropriate. Receiver Operating Characteristic (ROC) curve was used to define an IL-17A threshold above which patients would be considered positive. A Wilcoxon matched pairs signed rank test was used to compare two measurements of a quantitative variable performed on the same subjects. Statistical analyses were performed using GraphPad Prism 7.0 (GraphPad Software, Inc., San Diego, CA) or SAS 9.0. Differences were considered significant when *P* value < 0.05.

## Results

### Serum Cytokine Levels in MN Patients Compared to Healthy Subjects After *In Vitro* Stimulation of Immune Cells

#### Serum Cytokine Levels in MN Patients

Without *in vitro* stimulation, a very low level of all cytokines was detected in serum (for example, IL-17A: 40.94 pg/ml [IQR, 0–96] after stimulation by anti-CD3 and R848 *vs* 0 pg/ml [IQR, 0–0] without stimulation, *P*=0.0005; IFN-γ: 4418 pg/ml [IQR, 1248–10350] after stimulation by anti-CD3 and R848 *vs* 0 pg/ml [IQR, 0–0] without stimulation, *P*<0.0001) (**Supplemental Figure 2**). This experiment was carried out on 14 MN patients before the start of the study to confirm the relevance of stimulating immune cells.

#### *In Vitro* Stimulation of Innate Cells *Versus* T Cells

**Figure 1:**
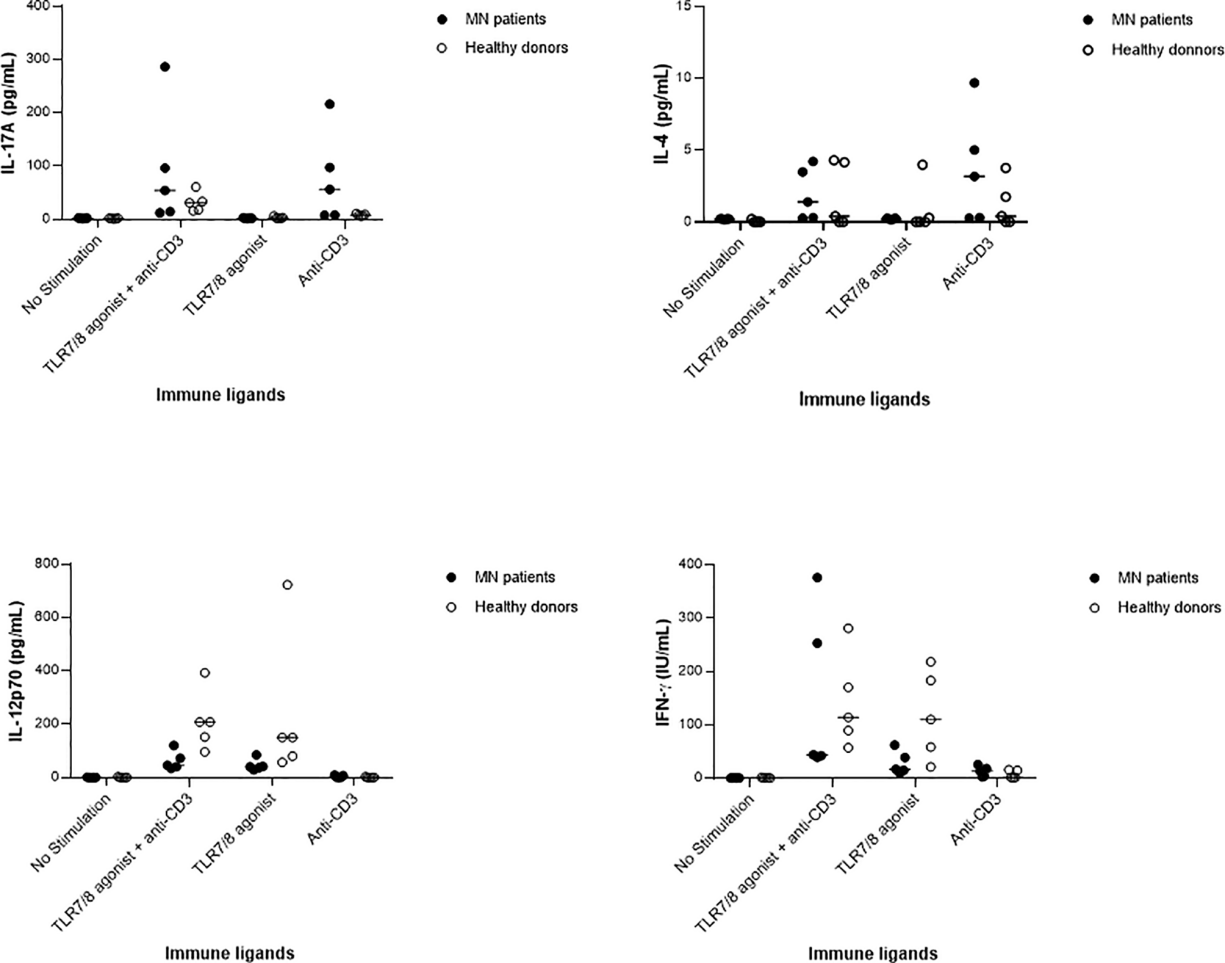
Serum cytokine levels in five membranous nephropathy (MN) patients and five healthy donors after *in vitro* stimulation of immune cells by anti-CD3, or TLR 7/8 agonist, or both anti-CD3 and TLR 7/8 agonist, or no stimulation. In both MN patients and healthy donors, the cytokines IL-17A and IL-4 were produced upon stimulation by anti-CD3, IL-12p70 as produced upon stimulation with TLR7/8 agonist, and the production of IFN-γ as increased after the stimulation of both innate and T cells.

Immune cells of 5 MN patients and 5 healthy donors paired for age (MN: mean age 45 ± 24 years *vs* HD: mean age 39 ± 16 years, *P*=0.63) and gender were stimulated with immune ligands: anti-CD3 as T-cells stimulant, TLR 7/8 agonist as a stimulant of macrophages and dendritic cells, or with both anti-CD3 and TLR 7/8 agonist. As shown in **Figure 1**, MN patients appeared to have increased levels of anti-CD3-induced IL-17A and IL-4, and impaired levels of IL-12p70 and IFN-γ after stimulation with the TLR7/8 agonist. This preliminary descriptive result suggests an enhanced Th17 and Th2 response and an impairment of the Th1 pathway in MN patients, as recently proposed by Li et al. (40).

#### Cytokine Levels in MN Patients After *In Vitro* Stimulation of Both Innate and T Cells

To approximate the pathophysiological conditions of a viral stimulation, described as a frequent immune trigger in MN, we induced *in vitro* stimulation of innate response by TLR7/8 agonist (47), and T lymphocytes by anti-CD3, in MN patients and controls, mimicking an activation of both innate and specific immune pathways. Fifty-nine patients with active MN (39 men and 20 women; mean age 53 ± 17 years) were enrolled, as well as 28 healthy donors (mean age 48 ± 14 years). For 26 MN patients who entered into remission follow-up samples were available. Patient characteristics are summarized in **Table 1**. Forty-two patients (71%) had anti-PLA2R1 antibodies, two (3%) had anti-THSD7A antibodies and 15 patients (26%) were negative for both anti-PLA2R1 and anti-THSD7A.

**Table 1:** Baseline characteristics of MN patients (*n*=59).

Characteristics	Value
**Age (years)**	53 ± 17
**Sex**	
Male	39 (66%)
Female	20 (34%)
**Etiology**	
Anti-PLA2R1-associated MN	42 (71%)
Anti-THSD7A-associated MN	2 (3%)
Double negative patients	15 (26%)
**Laboratory evaluations**	
UPCR (g/g)	4.29 [2.42 – 7.80]
PLA2R1-Ab titer (RU/ml)	43.5 [16.0 – 199.3]
Serum creatinine (µmol/L)	118.5 [86.0 – 230.0]
Albuminemia (g/L)	31.85 [21.88 – 36.75]
Urea (mmol/L)	8.65 [5.90 – 14.10]
Lymphocyte count (G/L)	1.7 [1.4 – 2.3]

Ab, antibody; MN, membranous nephropathy; PLA2R1, phospholipase A2 receptor 1; THSD7A, thrombospondin type-1 domain-containing protein 7A; UPCR, urine protein to creatinine ratio.

**Figure 2:**
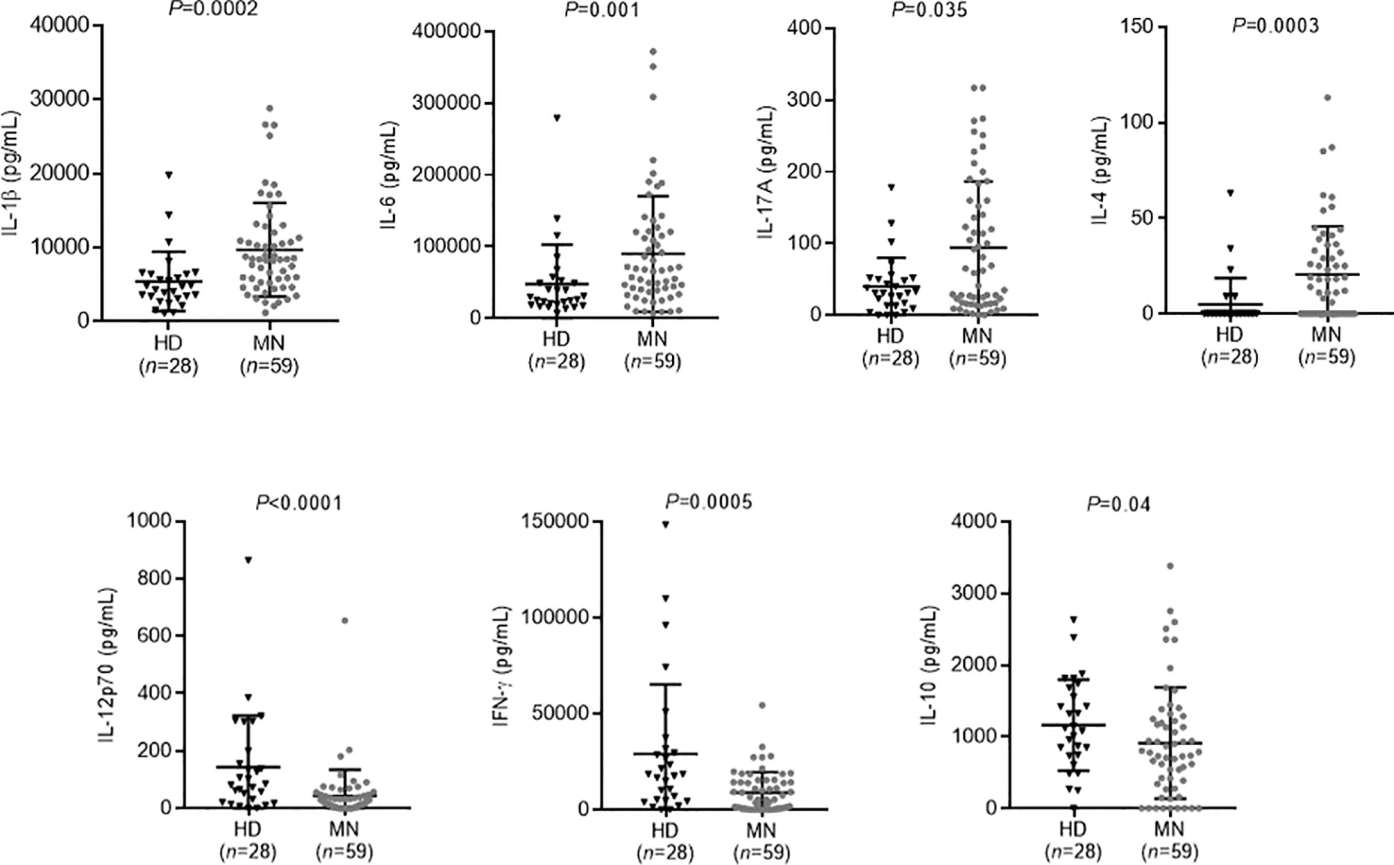
Serum cytokine levels in membranous nephropathy (MN) patients and healthy donors after *in vitro* stimulation of immune cells by both anti-CD3 and TLR 7/8 agonist. Compared to healthy donors, MN patients had increased levels of IL-1β, IL-6, IL-17A, and IL-4, and decreased levels of IL-12p70, IFN-γ, and IL-10. A non-parametric two-tailed test (Mann-Whitney) was used to compare the level of cytokines in patients with that in healthy donors. MN, membranous nephropathy; HD, healthy donors.

Supernatant IL-17A, IL-6, IL-1β, IFN-*γ*, IL-12p70, TNF-α, IL-10, IL-5, IL-4, IL-13, and GM-CSF were measured after *in vitro* stimulation. The concentration of inflammatory cytokines IL-1β and IL-6 implicated in innate immune response was significantly higher in MN patients than in the healthy control group (IL-1β: 8405 pg/ml [IQR, 5224–12065] versus 4522 pg/ml [IQR, 3183–6418], *P*=0.0002; IL-6: 66767 pg/ml [IQR, 36860–120978] versus 27979 pg/ml [IQR, 18672–51499], *P*=0.001). Th17 and Th2 cytokines IL-17A and IL-4, respectively, were also significantly increased in MN patients in comparison to healthy subjects (IL-17A: 62 pg/ml [IQR, 16–160] versus 31 pg/ml [IQR, 13–51], *P*=0.035; IL-4: 12 pg/ml [IQR, 0–33] versus 0 pg/ml [IQR, 0–0], *P*=0.0003) (**Figure 2**).

On the other hand, the concentration of Th1 cytokines IFN-γ and IL-12p70, as well as of a regulatory T cells cytokine IL-10 was lower in MN patients in comparison to healthy subjects (IFN-γ: 5320 pg/ml [IQR, 501–14325] versus 18037 pg/ml [IQR, 4889–31329], *P*=0.0005; IL-12p70: 23 pg/ml [IQR, 5–48] versus 83 pg/ml [IQR, 24–189], *P*<0.0001; IL-10: 778 pg/ml [IQR, 340–1247] versus 1102 pg/ml [IQR, 737–1652], *P*=0.04) (**Figure 2**).

There was no difference in TNF-α, IL-5, IL-13 and GM-CSF concentrations between MN patients and healthy subjects (*data not shown*).

These data suggest an activation of the Th17 and Th2 pathways and a deficit of the Th1 and Treg pathways in MN patients as observed in many autoimmune diseases (29, 30, 39).

### Th17 Profile and Air Pollutants

A threshold of 58 pg/ml was defined to distinguish the patients with positive IL-17A activity, determined using ROC analysis with a sensitivity of 86% and a specificity of 52% (AUC=0.64, *P*=0.035) (**Supplemental Figure 3**): 31 patients (53%) had a high level of IL-17A.

Forty-one patients (69%) lived in urban areas and 18 (31%) in rural areas. As previous studies demonstrated that exposure to PM_2.5_ induces production of pro-inflammatory cytokines (IL-1β and IL-6) (48) and is correlated with a higher risk of MN (3), we aimed to analyze the levels of air pollution according to the Th17 profile of MN patients.

**Figure 3:**
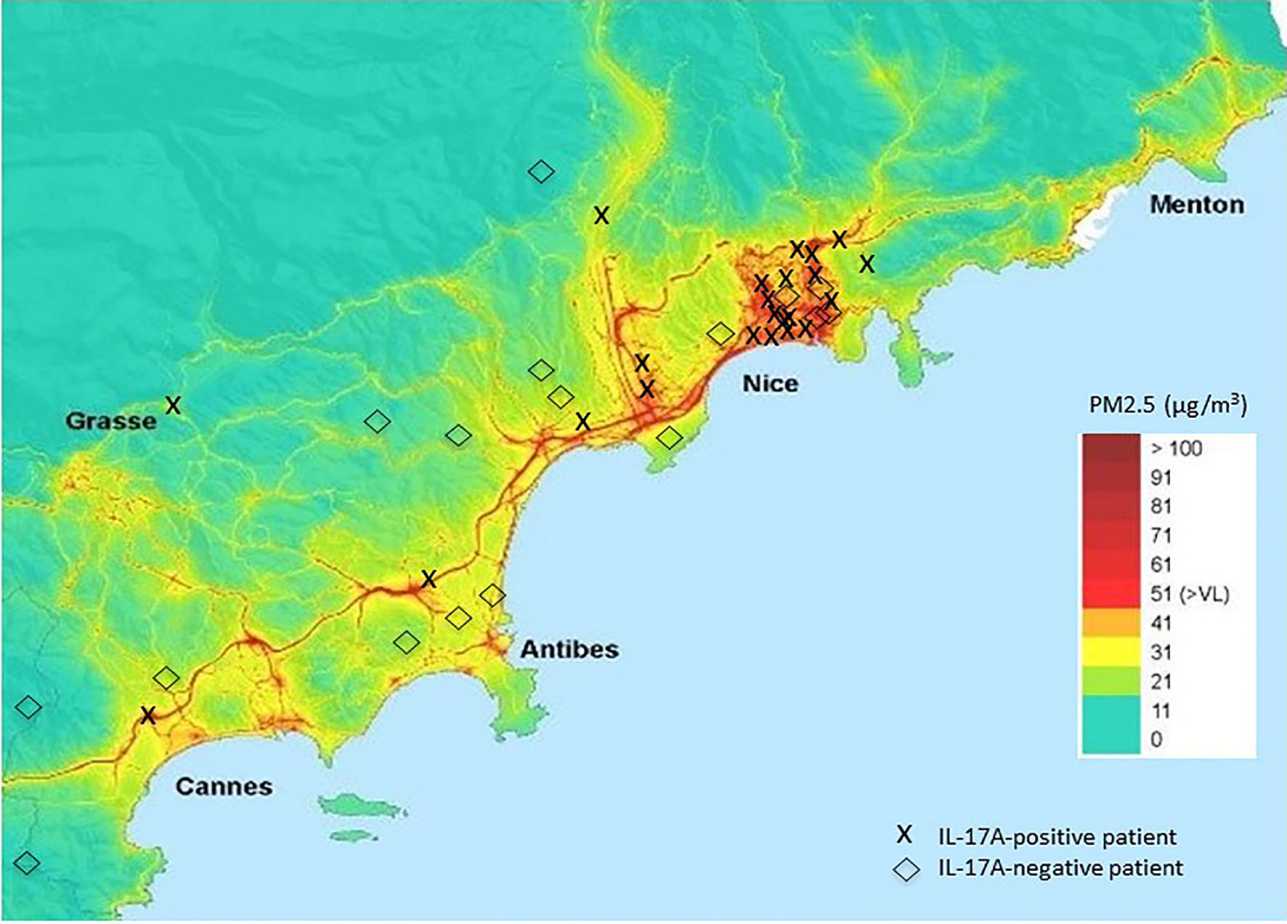
Superposition of the usual area of residence of membranous nephropathy (MN) patients and air pollution. Cumulative PM_2.5_ exposition in 2016 in the French region Provence-Alpes-Côte d’Azur is presented as heat map. The usual area of residence of each MN patient living in the French region Provence-Alpes-Côte d’Azur is represented by a cross (IL-17A-positive MN patients) or a rhombus (IL-17A-negative MN patients). IL, interleukin; PM, particulate matter.

IL-17A-positive patients were exposed to significantly higher levels of PM_2.5_ than IL-17A-negative patients at their usual area of residence: 51 µg/m^3^ [IQR, 46–51] versus 31 µg/m^3^ [IQR, 21-36], respectively (*P*=0.002) (**Figure 3**). The mean French exposition is evaluated at 12 μg/m^3^ and the World Health Organization recommends an exposition below 10 μg/m^3^ (49).

### Cytokine Profiles and Prognosis

#### Th17 Profile and Prognosis of MN

We then analyzed the impact of this inflammatory profile on MN prognosis. We found no difference in proteinuria, albuminemia, serum creatinine, urea, lymphocyte count, anti-PLA2R1 activity and remission between IL-17A-positive and IL-17A-negative patients but IL-17A positive patients presented more thromboembolic complications (phlebitis, pulmonary embolism, renal vein thrombosis) and relapsed more often (*P*=0.03 and *P*=0.0006, respectively, **Table 2**).

**Table 2:** Characteristics of MN patients according to interleukin-17A level.

	IL-17A-positive patients (*n*=31)	IL-17A-negative patients (*n*=28)	*P*
**Demography**			
Age (years)	54 ± 16	52 ± 19	0.66
Sex ratio (F/M)	9/21	11/17	0.58
**Laboratory evaluations**			
PLA2R1-Ab titer (RU/ml)	29 [8 – 271]	53 [19 – 95]	0.66
UPCR (g/g)	3.95 [1.89 – 6.59]	4.61 [3.53 – 8.15]	0.15
Serum creatinine (µmol/L)	123 [88 – 159]	117 [76 – 255]	0.86
Albuminemia (g/L)	34.5 [24.7 – 37.2]	30.3 [17.8 – 35.8]	0.32
Urea (mmol/L)	9.6 [5.9 – 13.0]	8.6 [6.2 – 15.4]	0.60
Lymphocyte count (G/L)	1.6 [1.4 – 2.5]	1.7 [1.2 – 2.1]	0.47
**Environmental data**			
PM2.5 level (µg/m3)	51 [46-51]^a^	31 [21-36]^b^	**0.002**
**Venous thromboembolic events**	12 (39%)	2 (7%)	**0.03**
**Remission at 6 months after rituximab treatment**			0.78
	Remission: 12/22	Remission: 8/16	
	No remission:10/22	No remission: 8/16	
**Relapses**	21	6	**0.0006**

Ab, antibody; MN, membranous nephropathy; PLA2R1, phospholipase A2 receptor 1; THSD7A, thrombospondin type-1 domain-containing protein 7A; UPCR, urine protein to creatinine ratio.

^a^Data was missing for two patients.

Bold values are p value statistically significant.

^b^Data was missing for six patients.

**Figure 4:**
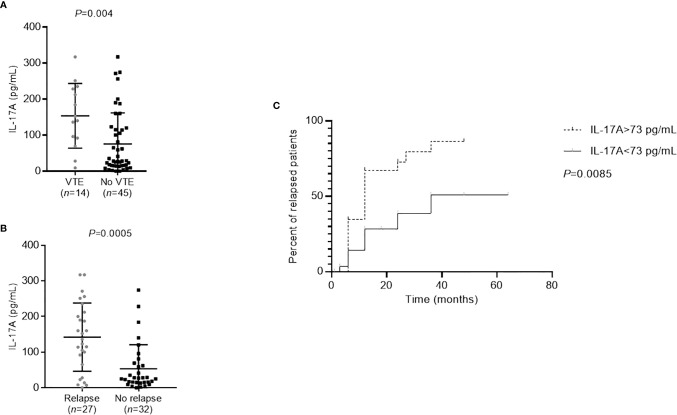
Prognosis of membranous nephropathy (MN) patients according to the level of cytokines. **(A)** Patients with thromboembolic complications (VTE) had significantly higher IL-17A levels than those without (*P*=0.004). Statistical significance was determined by a Mann-Whitney test. **(B)** Patients who relapsed within one year after rituximab treatment had significantly higher IL-17A levels than those who did not relapse (*P*=0.0005). Statistical significance was determined by a Mann-Whitney test. **(C)** Relapse-free survival was lower in patients with higher IL-17A levels at diagnosis. The threshold of 73 pg/ml, as determined by ROC curve (sensitivity of 81% and specificity of 76%), was used to distinguish the patients with low or high levels of IL-17A. Kaplan-Meier analysis was used to estimate the relapse-free survival of MN patients (*n*=59) based on their IL-17A level. IL, interleukin; MN, membranous nephropathy; VTE, venous thromboembolic event.

We then focused on patients who had thromboembolic complications or who relapsed within one year after rituximab treatment. These patients had significantly higher IL-17A levels than the patients without thromboembolic complications (*P*=0.004) and the patients who did not relapse (*P*=0.0005) (**Figures 4A, B**). An IL-17A threshold at 73 pg/ml was defined to identify patients at risk of relapse, using ROC curve with a sensitivity of 78% and a specificity of 75% (AUC=0.77, *P*<0.001, *data not shown*). Patients with more than 73 pg/ml of IL-17A were 10.50-times more likely to relapse (odds ratio=10.50 [IQR, 3.13–35.20]) and renal survival without relapse was significantly lower for these patients (*P*=0.0085) (**Figure 4C**). It is important to note that in our patient population with active MN but often non-nephrotic proteinuria, albuminemia and serum creatinine levels do not correlate with the occurrence of venous thromboembolic events (VTE) (albuminemia: 26.1 mg/L [18.7–35.3] in patients with VTE *vs* 33.0 mg/L [22.9–36.8] without VTE, *P*=0.21; serum creatinine: 111 µmol/L [88–143] in patients with VTE *vs* 124 µmol/L [81–253] without VTE, *P=*0.56) or relapses (albuminemia: 34.2 mg/L [27.0–36.8] in relapsing patients *vs* 26.2 mg/L [19.2–36.3] in non-relapsing patients, *P*=0.11; serum creatinine: 133 µmol/L [97–247] in relapsing patients *vs* 96 µmol/L [80–192] in non-relapsing patients, *P=*0.23).

#### Cytokine Profile of MN Patients in Remission

**Figure 5:**
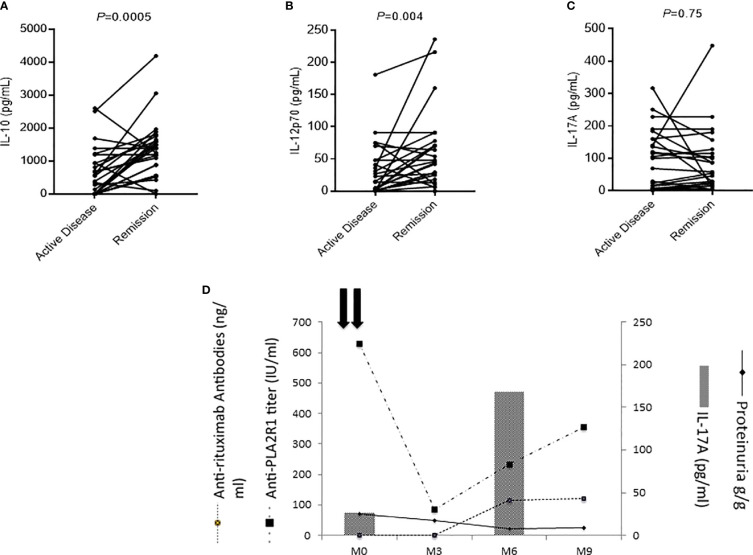
Evolution of membranous nephropathy (MN) patients according to the level of cytokines. **(A–C)** Evolution of serum cytokine levels in MN patients who entered into remission (*n*=26). Remission was associated with a significant increase of IL-10 **(A)** and IL-12p70 **(B)** levels, while the level of IL-17A remained unchanged **(C)**. A Wilcoxon matched pairs signed rank test was used to compare cytokine levels performed on the same MN patients during active disease and in remission. **(D)** Individual evolution of interleukin-17A levels of a MN patient according to his clinical evolution. This patient had low IL-17A levels before treatment with rituximab but developed antibodies against rituximab at month-6 associated with an increase of anti-PLA2R1 antibodies and an increase of IL-17A demonstrating a shift toward a Th17 profile.

We measured cytokine profile in the serum of 26 patients both in active phase of the disease and in remission induced by rituximab (1g at two-weeks interval). IL-10 and IL-12p70 levels increased at remission (IL-10: 467 pg/ml [IQR, 0–1005] in the active phase versus 1382 pg/ml [IQR, 798–1676] at remission, *P*=0.0005; and IL-12p70: 15 pg/ml [IQR, 0–48] versus 45 pg/ml [IQR, 17–73], respectively, *P*=0.004) (**Figures 5A, B**). No changes were observed for IL-17A (**Figure 5C**) suggesting that rituximab did not impact on Th17 profile.

#### Evolution of IL-17A Levels After Rituximab Treatment

During follow-up, IL-17A levels were stable during active disease and at remission for most patients (**Figure 5C**). One IL-17A-negative patient relapsed after rituximab treatment and developed anti-rituximab antibodies at the moment of the cytokine profile switch toward the Th17 pathway (IL-17A: 27 versus 168 pg/ml) (**Figure 5D**) (50). In this patient the onset of the Th17-mediated inflammation was thus associated with the immunization against rituximab, reappearance of anti-PLA2R1 antibodies and MN relapse (**Figure 5D**).

## Discussion

Membranous nephropathy is a rare autoimmune renal disease with an increasing prevalence. Previous studies showed a polarization toward Th2 immune response in idiopathic MN patients (35–37) (using RT-PCR) with little or no information on Th17 pathway cytokines, which are involved in the development of autoimmune diseases (29, 30). Rosenzwajg et al. observed a decrease of IL-17A in MN patients that could be related to urinary leakage of proteins in nephrotic patients (39) whereas Li and al. suggested an increased Th17 immune response detected by intracellular cytokine assay following stimulation with a leukocyte activation cocktail. (40). *In vitro* non-specific stimulation of lymphocytes and innate immunity cells prior to measuring cytokines allowed us to detect several cytokines in the serum of MN patients despite nephrotic range proteinuria. Our study showed a significant increase of pro-inflammatory cytokines (IL-1β and IL-6) and of Th2-related cytokines (IL-4) in MN patients compared to healthy subjects, as well as a decrease in Treg (IL-10) and Th1-related cytokines (IL-12p70 and IFN-γ), as previously shown (35–39). We have also shown an activation of Th17 pathway in MN patients, confirming GWAS data published by Xie et al. (27).

This polarization toward Th17 immune response in MN patients appears to be associated with an urban environment: MN patients with Th17-positive profile were more exposed to PM_2.5_. In accordance with our results, a Chinese team recently showed in a double-blind, randomized crossover study that higher PM_2.5_ exposure is positively associated with a higher level of cytokines IL-1β and IL-6 (48). Since a geographical correlation between the occurrence of MN and the satellite analysis of exposure to fine particles (PM_2.5_) has already been demonstrated (3), our results reinforce the hypothesis of an environmental origin of the onset of MN in a subset of patients exposed to air pollution with a Th17-profile. In addition, exposure to PM_2.5_ could also be considered as a danger signal which induces activation of autoreactive T lymphocytes, which may be a trigger required to induce an autoimmune response (51).

Clinically, serum IL-17A concentrations were associated with poor prognosis of MN defined by more thromboembolic complications and more relapses. The remission was heralded by a recovery of the Treg and Th1 pathways as previously described (38, 39). In contrast to Berti et al. who showed that IL-6 was reduced in response to rituximab during anti-neutrophil cytoplasmic antibody-associated vasculitis (52), in our study MN patients in complete remission maintained a Th17 profile and treatment with rituximab did not allow a reorientation of the Th17-mediated inflammatory response. The stability of the immune response toward the Th17 pathway despite remission and immunosuppressive therapy could be explained by the persistence of pro-inflammatory environmental triggers. These data suggest that the detection of Th17-mediated inflammation at diagnosis could raise the question of a treatment with anti-IL-6 or anti-IL-17A associated with rituximab.

This study has several limitations. First, it is limited to a single territory with a poor contrast between polluted and unpolluted areas. A large-scale study on a larger territory should be initiated to confirm the association between air pollution and the Th17 profile of MN patients. Second, we did not identify the environmental factor(s) associated with non-Th17 related MN in patients from rural areas. It would be interesting to study the role of certain more specific exposures of this population such as pesticides, wood combustion, the use of diesel engines etc. Third, this is an association study that cannot confirm the causal link between the urban environment, the modification of the cytokine profile of patients and the development of MN. *In vitro* studies on PBMC and podocytes, and *in vivo* studies on animal models are necessary to assess the impact of exposure to particulate matter on the cytokine profile. These three points are the objectives of a French hospital clinical research program called Immunopathological Analysis in a French National Cohort of Membranous Nephropathy (NCT04326218) which started including MN patients in July 2020.

In conclusion, 53% of MN patients have Th17-mediated inflammation appearing to be related to an urban environment. Clinically they have a poor prognosis defined by more thromboembolic events and relapses, and their Th17 cytokine profile remains unchanged after treatment with rituximab. Treatment inducing re-orientation of the immune system could be beneficial for these patients.

## Data Availability Statement

The original contributions presented in the study are included in the article/**Supplementary Material**. Further inquiries can be directed to the corresponding author.

## Ethics Statement

The studies involving human participants were reviewed and approved by French local etc committee NCT02199145. The patients/participants provided their written informed consent to participate in this study.

## Author Contributions

BS-P designed the study. VB and MC carried out experiments. MC, BS-P, VB, SP, and FD analyzed and interpreted the data. BS-P, VE, MC, SB-S, MA, and AG provided medical oversight. MC, BS-P, and VB drafted and revised the manuscript. All authors contributed to the article and approved the submitted version.

## Funding

This research was supported by grants from GIRCI-Méditerranée, ORKID and Conseil Départemental des Alpes-Maritimes.

## Conflict of Interest

Some co-authors are coinventors on the patents “Methods and kits for monitoring membranous nephropathy” (BS-P), and “Prognosis and monitoring of membranous nephropathy based on the analysis of PLA2R1 epitope profile and spreading” (BS-P, VE).

The remaining authors declare that the research was conducted in the absence of any commercial or financial relationships that could be construed as a potential conflict of interest.

## Supplementary Material

Supplementary Figure 1 Location of pollutants measuring stations in the French region Provence-Alpes-Côte d’Azur (PACA) (Atmosud data).

Supplementary Figure 2 Serum cytokine levels in MN patients with and without in vitro stimulation of immune cells by anti-CD3 and TLR 7/8 agonist (R848). No cytokines were detected in serum without in vitro stimulation of immune cells by anti-CD3 and TLR 7/8 agonist. A non-parametric two-tailed test (Mann-Whitney) was used to compare the level of cytokines. IL, interleukin; MN, membranous nephropathy.

Supplementary Figure 3 Receiver operating characteristic curve to distinguish IL-17A-positive patients and IL-17A negative patients. An IL-17A level greater than 58 pg/mL was associated with an IL-17A positivity (sensitivity of 86% and specificity of 52%). AUC, area under the curve; ROC, receiver operating characteristic curve.
