# Supplementary figures and images for: Th17-Immune Response in Patients With Membranous Nephropathy Is Associated With Thrombosis and Relapses
Source: Front Immunol. 2020 Nov 26;11:574997. doi: 10.3389/fimmu.2020.574997 (PMC7725714; doi:10.3389/fimmu.2020.574997)

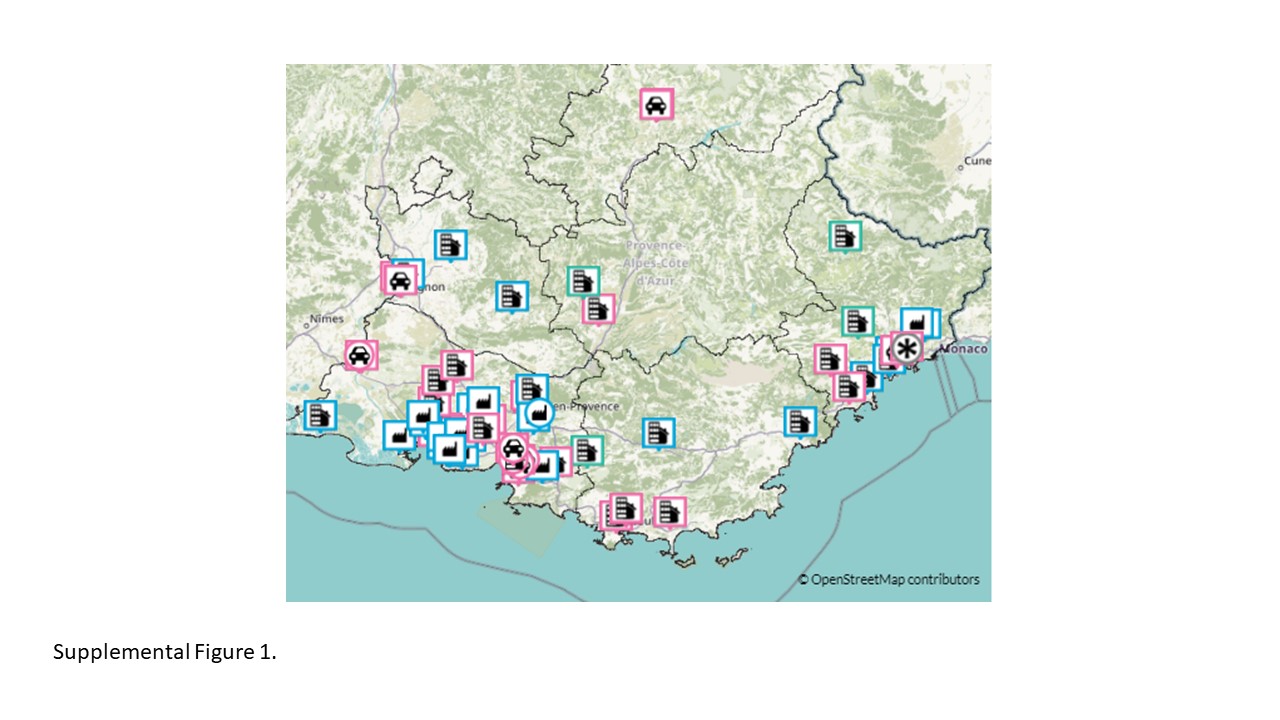

Supplement: Supplementary Figure 1 — Location of pollutants measuring stations in the French region Provence-Alpes-Côte d’Azur (PACA) (Atmosud data). [file Image_1.jpeg]

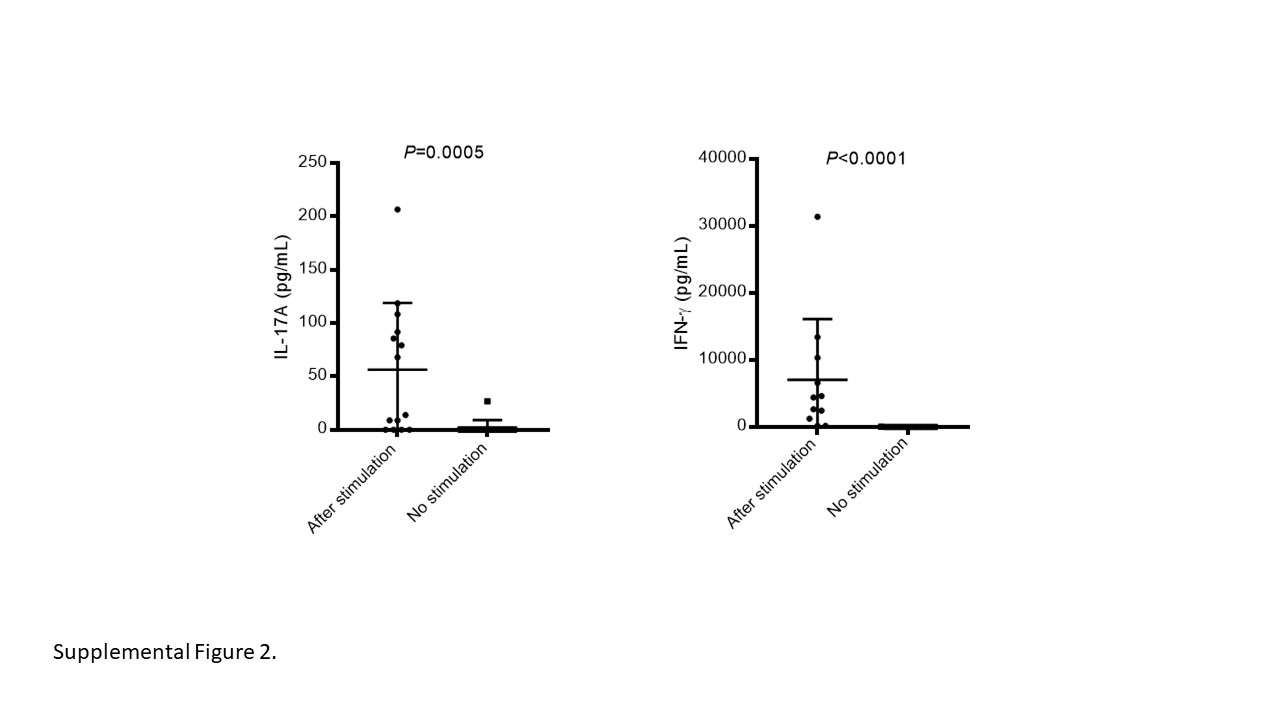

Supplement: Supplementary Figure 2 — Serum cytokine levels in MN patients with and without in vitro stimulation of immune cells by anti-CD3 and TLR 7/8 agonist (R848). No cytokines were detected in serum without in vitro stimulation of immune cells by anti-CD3 and TLR 7/8 agonist. A non-parametric two-tailed test (Mann-Whitney) was used to compare the level of cytokines. IL, interleukin; MN, membranous nephropathy. [file Image_2.jpeg]

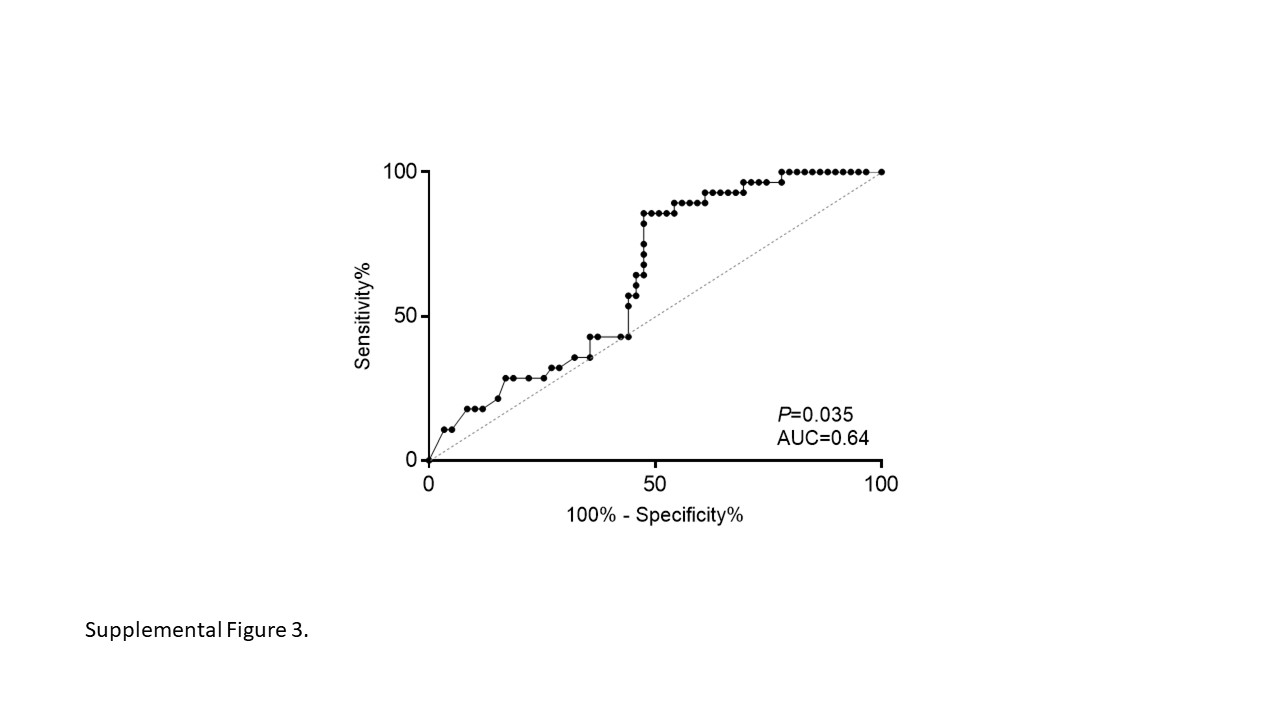

Supplement: Supplementary Figure 3 — Receiver operating characteristic curve to distinguish IL-17A-positive patients and IL-17A negative patients. An IL-17A level greater than 58 pg/mL was associated with an IL-17A positivity (sensitivity of 86% and specificity of 52%). AUC, area under the curve; ROC, receiver operating characteristic curve. [file Image_3.jpeg]
